# A multicenter study on the profile of fungal infections in children infected with HIV in tertiary hospitals in South Western Nigeria

**DOI:** 10.3389/fped.2026.1812083

**Published:** 2026-10-05

**Authors:** Olusola Adetunji Oyedeji, Joseph Babafunso Ayinde, Odunayo Oluseun Afolayan, Funso Abidemi Olagunju, Temitope Ajibola Oyelami, Ayobami Oyetunji Alabi, Olanrewaju Olayemi, Samuel Olorunyomi Oninla, Michaeline Asuquo Isawumi, Omomayowa Omotola Fawale, Adenike Oluwakemi Oladimeji, Olusola Ojurongbe

**Affiliations:** 1 Department of Paediatrics and Child Health, Osun State University Teaching Hospital, Osogbo, Nigeria; 2 Department of Paediatrics and Child Health, Obafemi Awolowo University Teaching Hospital Complex, Ife, Nigeria; 3 Department of Paediatrics and Child Health, Ladoke Akintola University of Technology, Ogbomoso, Nigeria; 4 Department of Paediatrics and Child Health, College of Health Sciences, Osun State University, Osogbo, Nigeria; 5 Department of Paediatrics and Child Health, College of Health Sciences, Ladoke Akintola University of Technology, Ogbomoso, Nigeria; 6 Department of Medicine, College of Health Scieneces, Osun State University, Osogbo, Nigeria; 7 Department of Surgery (Opthamology Unit), College of Health Sciences, Osun State University, Osogbo, Nigeria; 8 Department of Paediatrics and Child Health, Wesley Guild Hospital, Ilesa, Nigeria; 9 Department of Paediatrics and Child Health, University of Osun Teaching Hospital, Osogbo, Nigeria; 10 Center for Emerging and Re-emerging Infectious Diseases, Ladoke Akintola University of Technology, Ogbomosho, Nigeria

**Keywords:** coinfections, fungal, human immunodeficiency virus, infections, opportunistic

## Abstract

**Background:**

Fungal infections in children infected with human immunodeficiency virus (HIV) are a significant cause of morbidity and mortality. However, there are no reports on this subject in Nigerian children.

**Objective:**

This study aimed to document the spectrum of fungal infections and their outcomes in Nigerian children infected with HIV.

**Methodology:**

The case records of all children infected with HIV in three South West Nigerian tertiary hospitals were studied. Data on the identified fungal infection, clinical presentation, and outcomes were obtained and analyzed.

**Results:**

A total of 175 children infected with HIV, comprising 89 (50.9%) boys and 86 (49.1%) girls, with ages ranging from 2 months to 16 years, were studied. Of the population studied, 83 (47.4%) had dermatophytosis and 24 (13.7%) had invasive fungal infections (IFIs). Tinea capitis, thrush (pityriasis versicolor), and tinea corporis constituted more than 75% of the dermatophytosis. Invasive candidiasis, *Pneumocystis* pneumonia, cryptococcal disease, and aspergillosis accounted for more than 95% of the IFIs. Among the 175 children, 61 (34.9%) admissions and 12 (6.9%) deaths were recorded. Nine of the 12 deaths were due to IFIs. Infants and children not on Highly Active Anti-Retroviral Therapy had higher rates of IFIs (*p* < 0.01). Admission and death rates were higher among those with IFIs (*p* < 0.01). Higher rates of IFIs and dermatophytosis were found among those with a low socioeconomic status (*p* < 0.05). Appropriate diagnostics were lacking for *Pneumocystis* pneumonia and cryptococcal disease diagnosis. Fluconazole was the only antifungal available and accessible for cryptococcal meningitis management.

**Conclusion:**

Fungal infections cause significant morbidity and mortality in children infected with HIV. Appropriate diagnostics and medications must be made accessible to reverse IFI treatment outcomes.

## Introduction

Fungal and human immunodeficiency virus (HIV) coinfection is common in the majority of sub-Saharan African countries with high HIV infection rates and high rates of fungal infections. Available reports show that between 5% and 11.8% of Africans are infected with serious fungal infections, with yearly estimates for superficial fungal infections much higher than those for serious fungal infections (1–4). Prevalence rates of diagnosed and undiagnosed HIV in African children are significant (2, 5). Nigeria, the most populous black country with a population of over 270 million, is bound to have a high coinfection rate for HIV and fungal infection. Defining this burden is expedient and it is a prerequisite for both control and prevention.

Serious fungal infections are significant contributors to admissions and death in individuals infected with HIV in sub-Saharan Africa (1–4). Predisposition to opportunistic infections is a common consequence of the compromised immune system in individuals with advanced HIV (6–8). However, surprisingly, the fungal infections responsible for opportunistic infections in individuals infected with HIV are underreported and information on their burden and outcomes in children in sub-Saharan Africa is scarce (8). Possible reasons adduced for this underreporting in previous studies include a lack of essential diagnostics and equipped health facilities and a shortage of trained personnel to diagnose and appropriately manage affected individuals in sub-Saharan Africa (6, 7).

There is a knowledge gap on fungal infections in Nigerian children infected with HIV. A literature review on this topic yielded only one study conducted in adults (9). This knowledge gap stimulated our interest in conducting this research and documenting the profile of fungal infections and outcomes in children infected with HIV who attended three tertiary hospitals in South West Nigeria. This study will provide information that will assist health-care providers and planners in doing their work more effectively. We also envisage that the results obtained in this pioneering study will stimulate interest in this subject and generate findings in related areas that require research.

## Methods

### Study participants and ethical approval

This study was a retrospective review of children infected with HIV enrolled for care at three different tertiary hospitals in Nigeria, namely, Wesley Guild Hospital (WGH), Ilesa; Osun State University of Osun (UNIOSUN) Teaching Hospital, Osogbo; and Ladoke Akinola University of Technology Teaching Hospital (LAUTECH), Ogbomoso. The case records of all children infected with HIV who were enrolled and receiving care in the three tertiary hospital pediatric antiretroviral units between 1 January 2010 and 31 December 2025 were studied and the necessary information was extracted. Ethical approval was obtained from the Ethics Review Committee of the Obafemi Awolowo University Teaching Hospitals Complex, University of Osun Teaching Hospitals, and Ladoke Akintola University of Technology Teaching Hospital. All the rules governing ethical research were observed and complied with in conducting this study. The ethical clearance certificate issued had the following protocol numbers: ERC/2025/08/05 from WGH, UTH/REC/2025/08/1302 from UNIOSUN Teaching Hospital, and LTH/OGB/EC/2025/678 from LAUTECH. Consent was also obtained from the health records unit to review the case notes. Sociodemographic data were extracted from the case notes, such as gender, age of the child at the last birth date, and parental occupation and highest educational status.

### Diagnosis of HIV infection

Diagnosis of HIV in all the subjects was confirmed by an appropriate serological positive retrovirus test report for all children aged older than 18 months and a positive DNA PCR test in children and infants younger than 18 months (10).

### Diagnosis of fungal injection

Additional details obtained from the case notes include the presence or absence of fungal infections, such as candidiasis, tinea infection, Pityriasis versicolor, Aspergillus infection, *Pneumocystis* pneumonia, and Cryptococcal infection, in the case records. Details confirming the diagnoses of the fungal infections were ascertained. The case definition of dermatophytosis was based on a characteristic history of an itchy rash appearance with response to the appropriate antifungal medication (9, 10). For example, the general definition of a tinea infection was an itchy oval rash with central clearing that resolved with ketoconazole or griseofulvin therapy. Oral thrush was characterized by a whitish adherent furring of the tongue or buccal mucosa surface that cannot be removed by light to firm pressure or scraping with a spatula (9). Candiduria was diagnosed microscopically by the presence of *Candida albicans* in the clean catch urine sample of an individual with symptoms of a urinary tract infection (UTI), with resolution of symptoms following appropriate daily doses of fluconazole therapy (11) and no resolution of the symptoms with conventional empiric antibiotics for a UTI.

The case definition of *Pneumocystis* pneumonia was based on findings of difficulty breathing and hypoxemia in infants infected with HIV who did not respond to the conventional antibiotic treatment for community-acquired pneumonia but who did respond to cotrimoxazole (9, 12). *Pneumocystis* pneumonia is associated with radiographic changes, such as interstitial infiltrates and mediastinal shadows from lymph node enlargement or ground-glass appearance. However, the expertise to isolate *Pneumocystis jirovecii* from bronchoalveolar lavage fluid was lacking in our facilities. The diagnosis of cryptococcal meningitis was based on the WHO recommendations for resource-restricted settings, namely, the identification of symptoms and signs of meningitis with a positive cryptococcal antigen antibody test of blood or cerebrospinal fluid (CSF) samples (13). India ink staining of the CSF for microscopic identification of cryptococcal organisms is the standard for proper diagnosis of cryptococcal meningitis (14, 15). This was not available in our setting, but a reliable diagnosis could still be made from the presentation and investigations based on recommendations for resource-restricted settings (13, 16). Radiographic features of cryptococcal pneumonia include reticulonodular or nodular or interstitial infiltrates (13, 14). The resolution of cryptococcal infection symptoms following administration of appropriate antifungals was also supportive of a correct diagnosis. A diagnosis of pulmonary aspergillosis was based on radiological evidence and detection of Aspergillus in the patient’s sputum sample on microscopy. *Cutaneous aspergillosis* was confirmed using microscopic and microbiological tests on skin biopsy samples by a dermatologist (17). In addition, the extra-ocular compartment of the eye and the fundus were examined by an ophthalmologist when indicated to determine if there was retina or external eye involvement.

A diagnosis of tuberculosis was based on a positive GeneXpert test with supportive radiological findings such as pulmonary cavities, hilar or mediastinal widening, and miliary shadows. A background history of prolonged fever, weight loss, or cough was also supportive in establishing a tuberculosis diagnosis. A malaria diagnosis was based on findings of the malaria parasite on the blood film in a febrile child, while the diagnosis of septicemia was based on clinical findings of fever with features of systemic involvement and isolation of bacteria in the blood culture of ill children. The presence of an exanthema or enanthem with a history suggestive of a viral origin in the affected child suggested the diagnosis of a viral infection. A diarrhea diagnosis was made if there was frequent passage of loose stools at a frequency more than the usual rate in the affected child. No autopsies were performed for any of the deaths, as all the parents refused an autopsy.

HIV infection staging was based on the WHO clinical staging (18, 19). The children’s outcomes were classified as discharged well and alive, discharged against medical advice, referred, or death. Socioeconomic classification was based on Oyedeji et al.’s method (20).

### Statistical analysis

All the data obtained from the extracted information were input into SPSS version 20 (IBM, USA) (21). Continuous variables are presented as means and ranges. Categorical data were analyzed using the chi-square and the *P*-value was deemed significant below 0.05.

## Results

### Distribution of the enrolled children based on the facilities at which care was received

A total of 175 children were enrolled in this study. In total, 21 children infected with HIV were enrolled and received HIV care at the pediatric HIV unit at Ladoke Akintola University of Technology, 70 children were enrolled for pediatric HIV care at Wesley Guild Hospital, and 84 were enrolled and treated for HIV at University of Osun Teaching Hospital.

### Age, sex, and social distribution of the children

The age of the children studied ranged from 2 months to 16 years, with a mean age of 9.0 ± 4.98 years. The gender distribution of the population was 89 (50.9%) boys and 86 (49.1%) girls. Concerning the socioeconomic status of the 175 children, 26 (14.8%) were from the upper socioeconomic groups, 44 (25.1%) were from the middle socioeconomic class, and 105 (60.0%) were from the lower socioeconomic groupings.

### WHO clinical stages of the children studied

The majority of the children were categorized as WHO clinical stage 2. Of the 175 children, 14 (8.0%) were categorized as clinical stage 1, 74 (42.3%) as clinical stage 2, 52 (29.7%) as stage 3, and 35 (20.0%) as stage 4.

### General admissions and illnesses in the studied children

Of the 175 children studied, 61 (34.9%) had a previous admission, while 115 (65.7%) had never been admitted. The indications for admission among those who were admitted are shown in Table 1. Other notable diseases recorded among those who had not been admitted include a case each of gastroenteritis with dehydration, umbilical hernia, chronic secretory otitis media, and pulmonary tuberculosis disease, which were managed on an outpatient basis.

**Table 1:** Indications for admission in the studied population.

Disease	Number	Percentage
Tuberculous disease	17	27.9
Candidiasis	15	24.6
Bronchopneumonia	6	9.8
Malaria with severe anemia	5	8.2
Chronic diarrhea	4	6.6
Lobar pneumonia	4	6.6
*Pneumocystis* pneumonia	3	4.9
Aspergillosis	2	3.3
Septicemia	2	3.3
Osteomyelitis	1	1.6
Cytomegalovirus retinitis	1	1.6
Diphtheria	1	1.6

Of the 17 cases of tuberculosis, 14 were pulmonary tuberculosis, two were tuberculous meningitis, and one was Pott’s disease. Of the 15 invasive candidiasis cases, 13 were esophageal candidiasis and there was one case each of candiduria and pulmonary candidiasis.

### Deaths

Twelve deaths were recorded among the 175 children, accounting for 6.9% of the total population studied. The cause of death was esophageal candidiasis, tuberculosis, *Pneumocystis* pneumonia, cryptococcal meningitis, and diphtheria, in five, two, two, two, and one case, respectively. Thus, of the 12 deaths recorded in this study, 9 (75.0%) were due to invasive fungal infections (IFIs). All the cryptococcal cases ended in fatality, while two (66.7%) of the three *Pneumocystis* pneumonia cases died and five (38.5%) of the esophageal candidiasis cases died. All the *Pneumocystis* pneumonia and cryptococcal cases occurred in infancy.

#### Challenges in diagnosis and management

Diagnoses of *Pneumocystis* pneumonia and cryptococcal pneumonia could not be made before 72 h of admission, because this time interval was needed to obtain the necessary results that provide the evidence. The non-availability of India ink at the hospital where the two children with cryptococcal meningitis were treated prevented an etiologic diagnosis. An etiologic diagnosis of *Pneumocystis* pneumonia could not be made because of the unavailability of trained personnel and appropriate equipment to obtain bronchoalveolar fluid for staining and microscopy. Also, treatment of cryptococcal disease was with parenteral fluconazole monotherapy, because of the unavailability of Amphotericin B and flucytosine. One of the patients who had been successfully managed and discharged for *Pneumocystis* pneumonia on cotrimoxazole therapy for 2 weeks relapsed 1 week after discharge and died after not responding to full doses of cotrimoxazole after readmission for 4 days.

### Pattern of fungal infections recorded in the children

The infections were divided into two broad classes, namely, dermatophytosis and invasive fungal infections. Of the 175 children studied, 83 (47.4%) had dermatophytosis and 92 (52.6%) did not. The pattern of dermatophytosis is presented in Table 2.

**Table 2:** Pattern of dermatophytosis among the studied children.

Dermatophytosis	Number	Percentage
Tinea capitis	23	27.7
Thrush	16	19.3
Pityriasis versicolor	14	16.9
Tinea corporis	10	12.1
Seborrheic dermatitis	3	3.6
Cutaneous candida	3	3.6
Tinea incognito	3	3.6
Moniliasis	2	2.4
Perineal candidiasis	2	2.4
Generalized tinea	2	2.4
Onychomycosis	2	2.4
Tinea pedis	1	1.2
Dhobie itch	1	1.2
Tinea facei	1	1.2
Total	83	100

In contrast, of the 175 children, 24 (13.7%) had invasive fungal infections and 151 (86.3%) did not. Of the 24 children with invasive infections, one had two invasive infections, thus accounting for a total of 25 invasive infections.

The 25 invasive infections comprised esophageal candidiasis, *Pneumocystis* pneumonia, cryptococcal disease, aspergillosis, Coccidioides immitis, candiduria, and pulmonary candidiasis and occurred in 15 (60.0%), 3 (12.0%), 2 (8.0%), 2 (8.0%),1 (4.0%),1 (4.0%) and 1 (4.0%) child, respectively.

### Association between dermatophytosis and some sociodemographic and clinical characteristics in the studied population

The majority of the children were in the 11–17 years age bracket; however, the majority of the superficial fungal infections occurred in the 6–10 years age bracket. Full details of the association between age group and the occurrence of dermatophytosis can be found in Table 2.

The total number of boys was slightly higher than the number of girls. The occurrence of superficial skin fungal infections was slightly higher in the boys compared to the girls, although this difference was not significant. Additional details can be found in Table 2.

The proportion of the studied population with superficial skin infections was higher among those who were admitted or died compared to those who were not admitted or survived, respectively. However, the differences between the two groups were not significant. The details can be found in Table 3.

**Table 3:** Associations between age, gender, social class, WHO clinical stage, admissions, outcome, and presence or absence of dermatophytosis.

	Dermatophytosis present (83)	Dermatophytosis absent (92)	*X* ^2^	*P*
Age				
1 day–5 years	24	26	3.98	0.14
6–10 years	31	23		
11–17	28	43		
Gender				
Male	44	45	0.29	0.59
Female	39	47		
Admissions				
Admitted	33	28	1.67	0.20
Not admitted	50	64		
Outcome				
Survived	77	86	0.34	0.85
Death	6	6		
WHO stage				
Non-advanced	38	50	1.28	0.26
Advanced	45	42		
Social class				
Upper	7	19	5.15	0.04
Lower	76	73		
Use of HAART				
Yes	58	68	0.35	0.55
No	25	24		

WHO, World Health Organization; HAART, Highly Active Anti-Retroviral Therapy.

A greater proportion of children infected with HIV from the lower socioeconomic classes and those with advanced HIV disease had higher rates of superficial fungal skin infection compared with their counterparts in the upper socioeconomic class and those without advanced HIV disease, respectively. The differences between the two groups were statistically significant and are shown in Table 3.

Concerning the use of Highly Active Anti-Retroviral Therapy (HAART), a greater proportion of children on HAART did not have superficial fungal skin infections, while slightly more than half of those not on HAART had dermatophytosis. The differences across the two groups were not significant and the full details on this association can be found in Table 3.

### Associations between invasive fungal infections and some clinical and social characteristics in the studied population

The majority of the invasive fungal infections were recorded in children aged less than 1 year, with significantly lower rates of invasive fungal infections in all the different age brackets above 1 year. The full details of the association can be found in Table 4.

**Table 4:** Association between invasive fungal infections and some clinical and social characteristics.

	Invasive fungal infections (*N*=24)	Non-invasive fungal infections (*N*=151)	*X* ^2^	P
Age				
<1 year	6	8	10.92	<0.01
1–5 years	3	32		
6–10	5	50		
≥11years	10	61		
Gender				
Male	10	79	0.94	0.33
Female	14	72		
Admissions				
Admitted	16	45	12.39	<0.01
Not admitted	8	106		
Outcome				
Survived	16	147	30.53	<0.01
Death	8	4		
WHO stage				
Non-advanced	1	87	23.67	<0.01
Advanced	23	64		
Social class				
Upper	0	26	4.85	0.03
Lower	24	125		
Use of HAART				
Yes	8	118	20.63	<0.01
No	16	33		

WHO, World Health Organization; HAART, Highly Active Anti-Retroviral Therapy.

A slightly higher rate of invasive fungal infection was found among girls, although this difference was not significant. The details can be found in Table 4.

Significantly higher rates of admissions and deaths were recorded among the children infected with HIV and invasive fungal infections. Full details of the association can be found in Table 4.

Significantly higher rates of fungal infection were found among children infected with HIV from the lower socioeconomic groups and among those with advanced HIV disease. Additional details concerning these associations can be found in Table 4.

Of the 126 children on HAART, eight had invasive fungal infections. Of the 49 who were not on HAART, 16 had invasive fungal infections. The difference between the two groups was statistically significant and full details can be found in Table 4.

### Atypical presentations of fungal infections

The majority of the fungal infection presentations among the children were typical. All the patients diagnosed with esophageal candidiasis had evidence of thrush. Five of the children with esophageal candidiasis presented with achalasia when swallowing food, in addition to thrush. Pulmonary candidiasis presented as a fever, difficulty breathing, and expectoration of sputum containing *Candida*, which was identified microscopically. Moreover, there was resolution of pulmonary shadows with fluconazole therapy on chest radiography. Three of the children with tinea corporis presented with tinea incognito, without annular lesions with central clearing and raised edges. However, a resolution or cure was achieved with daily oral griseofulvin therapy administered for 2 months.

Moniliasis was due to pregnancy in one of the adolescent girls and underlying diabetes mellitus in the remaining adolescent girl. It is also interesting to note that a girl with moniliasis also developed candiduria.

All the cases of *Pneumocystis* pneumonia presented with perihilar shadows and apically concentrated infiltrates with pneumatoceles. None of the cases presented with a ground-glass appearance.

### Immunological status of patients studied

The CD4 counts in the population without dermatophytosis or invasive fungal infection ranged from 761 to 1,758 cells/mm^3^, with a mean of 1,286.8 ± 431.3 cells/mm^3^. The CD4 counts for the population with dermatophytosis ranged from 345 to 742 cells/mm^3^, with a mean of 295.0 ± 350.4 cells/mm^3^. The CD4 counts of the patients with invasive fungal infections ranged from 11 to 343 cells/mm^3^, with a mean of 133. 0 ± 182.7 cells/mm^3^.

## Discussion

Fungal infections are common among children infected with HIV. The present study recorded notable superficial and invasive fungal infections, with the superficial fungal infections being three times as prevalent as invasive fungal infections. Our prevalence estimate of 47.4% for superficial fungal skin infections in this study at least doubles the majority of other estimates for cutaneous infections in children infected with HIV in Africa (22–24). Our higher prevalence estimate may be due to our focus on fungal infections compared to other studies that were focusing on general skin infections.

The predominance of superficial fungal infections in the present study is consistent with the findings of Oladele et al.’s study, which was conducted in Lagos, Nigeria (9). However, tinea capitis was the leading dermatophyte infection in our study, followed closely by thrush, contrasting sharply with Oladele et al.’s study, which reported thrush as the most prevalent infection. The difference in the results can be explained by the differences in the age distribution of the studied populations, as Oladele et al.’s study population was exclusively adults who are protected against tinea capitis at transition to puberty, when there is an increase in sebum production, sweat, and width of the hair (25). Secretion of sebum and postcolonization by Pityrosporum orbiculare have been found to protect against dermatophytic invasion of the scalp at puberty.

The prevalence and pattern of dermatophytosis in this study are similar to findings in previous studies in children infected with HIV, with the prevalence estimates ranging from 20.9% to 50.7% (22–24, 26). Our finding of a significantly greater rate of dermatophytosis among the lower socioeconomic classes is consistent with findings from other reports, as expected (27–29).

However, it is interesting to note that the prevalence and pattern of fungal infections in our study are similar to findings of studies on apparently healthy schoolchildren. These findings also mirror findings from a general pediatric dermatology clinic, thus indicating that dermatophytosis in children infected with HIV may not necessarily be opportunistic infections (27–30).

The predominance of dermatophytosis among the boys and children younger than 10 years in this study is consistent with previous reports (22–24), although these associations only attained clinical significance. The predominance of recorded tinea infections in the prepubertal age group has already been explained by the weighty contribution of tinea capitis before puberty (25).

Our findings of atypical tinea incognito presentation need to be highlighted, as this has not been previously reported in previous studies. Our explanation for the tinea incognito presentation as a fungal skin infection is that this is a sort of cutaneous allergy to the HIV infection. Healthcare providers need to be aware of the presentation of tinea incognito, as this will help reduce the occurrence of missed or incorrect diagnoses and mismanagement.

The challenge posed by unavailable diagnostics in resource-restricted settings was highlighted in this study (6, 7, 16). Fungal cultures were not conducted for the superficial and invasive fungal infections for two reasons. First, fungal studies are expensive, with the cost of culturing and identification of fungus from skin scrapings or skin biopsy costing up to $26, which is more than half the monthly salary of those on a minimum wage in Nigeria. Thus, a cost-effective option, namely empiric management, was the modus operandi and our confirmation of a correct diagnosis was based on the morphology of the superficial fungal infections’ presentations and their response to appropriate antifungals (9).

Invasive fungal infections were, however, more challenging to diagnose because of the invasive nature of the disease. Special training and equipment are needed to view and take samples from the site of infection for fungal studies (6, 7, 16). However, a correct diagnosis was still confidently made for esophageal candidiasis. In the same vein, a lack of trained personnel to obtain culture samples for fungal studies from bronchoalveolar lavage fluid prevented comprehensive investigations for *Pneumocystis* pneumonia, while comprehensive investigations for cryptococcal meningitis were hampered by a lack of appropriate equipment to measure the opening CSF pressure and India ink stain for CSF analysis. The challenges in the management of cryptococcal meningitis were further compounded by the inability to obtain parenteral amphotericin B and flucytosine; thus, the fatal outcomes were not unexpected.

Concerning other unusual pediatric fungal presentations, such as moniliasis, there is a need to both treat them aggressively and be aware of underlying conditions, such as pregnancy or diabetes mellitus, as evidenced in the present study. Aggressive treatment is necessary to forestall the development of other unusual invasive infections, such as candiduria, which was a complication in the present study. Untreated fungal infections can disseminate or progress if not promptly and appropriately managed, and may be the pathway from oral thrush to esophageal candidiasis.

The pattern of invasive fungal infections in the present study is similar to that reported by Oladele et al. (9), with esophageal candidiasis, *Pneumocystis* pneumonia, disseminated aspergillosis, and both systemic and cryptococcal meningitis accounting for almost all of the invasive fungal infections. These diseases are acquired immune deficiency (AIDS)-defining illnesses (31), and notable contributors to admission. All the cryptococcal and *Pneumocystis* pneumonia infections occurred in infants. The predominance of these invasive fungal infections in the infantile age bracket may be related to their naive or inexperienced immune system, coupled with a reduced CD4 lymphocyte count in those with invasive fungal infections.

The mortality rate for invasive fungal infections in the present study was higher than the 1.6% reported by Oladele et al. (9). Esophageal candidiasis, cryptococcal meningitis, and *Pneumocystis* pneumonia were the three invasive fungal infections with unacceptably high case fatality rates, accounting for all the fungal deaths and 75% of the total number of deaths recorded in this study. Difficulties in accessing the necessary diagnostics and appropriate treatment in a timely fashion were the main reasons for these high mortality rates. Drug testing for the fungi could not be conducted, since the fungi could not be isolated. Thus, the risk of resistance to the empirically administered antifungals is a possibility that cannot be ruled out.

This study also demonstrates the protective effect of Highly Active Anti-Retroviral Therapy for invasive fungal infections. The protective effect was statistically significant for the IFIs, but only of clinical significance for dermatophytosis. Several studies have shown the protective effect of HAART and the need to initiate HAART early while ensuring adherence in individuals infected with HIV (31–33). Notable organizations involved in the care of HIV that provide guidelines for the management and prevention of opportunistic infections also emphasize the impact of HAART on the prevention and treatment of opportunistic infections (33).

The immunological status of the children based on their CD4 lymphocyte count ranged from severe immunological deficiency to immune competence. The group of children without infections had the highest mean CD4 count. The mean CD4 count was lowest among those with invasive fungal infections, with the level indicating severe immunodeficiency, which explains why this group had severe, invasive, and systemic infections. In contrast, the CD4 counts in the children with dermatophytosis ranged from mild to moderate or no suppression, thus explaining the associated superficial fungal skin infections suffered by the children in this group. The presence or absence of a fungal infection and its severity were reflected in the CD4 lymphocyte count and the immunologic status of the studied subjects (34–36).

We conclude that dermatophytosis is very common among children infected with HIV and invasive fungal infections are a very significant cause of mortality among these children. Strategies that will uplift the socioeconomic status of the citizenry and increase the use of HAART among children infected with HIV with fungal infections should be engaged in order to positively impact patient outcomes.

## Data Availability

The raw data supporting the conclusions of this article will be made available by the authors, without undue reservation.
